# Environmental Adversity and Children’s Early Trajectories of Problem Behavior: The Role of Harsh Parental Discipline

**DOI:** 10.1037/fam0000258

**Published:** 2016-12-15

**Authors:** Eirini Flouri, Emily Midouhas

**Affiliations:** 1 Department of Psychology and Human Development, UCL Institute of Education, University College London

**Keywords:** adversity, internalizing and externalizing problems, Millennium Cohort Study, parenting, socioeconomic disadvantage

## Abstract

This study was performed to examine the role of harsh parental discipline in mediating and moderating the effects of environmental adversity (family socioeconomic disadvantage and adverse life events) on emotional and behavioral problems across early-to-middle childhood. The sample included 16,916 children (48% female; 24% non-White) from the U.K.’s Millennium Cohort Study. We analyzed trajectories of conduct, hyperactivity, and emotional problems, measured at ages 3, 5, and 7 years, using growth curve models. Harsh parental discipline was measured at these ages with parent-reported items on the frequency of using the physical and verbal discipline tactics of smacking, shouting at, and “telling off” the child. As expected, family socioeconomic disadvantage and adverse life events were significantly associated with emotional and behavioral problems. Harsh parental discipline was related to children’s trajectories of problems, and it moderated, but did not explain, the effect of environmental risk on these trajectories. High-risk children experiencing harsh parental discipline had the highest levels of conduct problems and hyperactivity across the study period. In addition, harsh parental discipline predicted an increase in emotional symptoms over time in high-risk children, unseen in their counterparts experiencing low levels of harsh parental discipline. However, children in low-risk families were also negatively affected by harsh parental discipline concurrently and over time. In conclusion, harsh parental discipline predicted emotional and behavioral problems in high- and low-risk children and moderated the effects of family poverty and adversity on these problems.

Much research finds that environmental adversity, such as family socioeconomic disadvantage (SED) and stressful life events, is linked with increases in children’s emotional (internalizing) and behavioral (externalizing) problems (Bradley & Corwyn, 2002; Drukker, Kaplan, Feron, & van Os, 2003; Goodnight et al., 2012; Kohen, Leventhal, Dahinten, & McIntosh, 2008). Harsh parental discipline (HPD; including harsh verbal discipline or physical punishment such as smacking) is also a powerful risk factor of child internalizing and, particularly, externalizing problems (Gershoff, 2002). HPD has interested prevention and intervention research because it also mediates (explains) the effect of parental depression on child problem behavior (Leung & Slep, 2006) and is related to some other well-established proximal risk factors of child problem behavior, such as young parental age, family dysfunction, and parental delinquency history (Jansen et al., 2012). Although there is evidence that HPD is also a response to (as well as an antecedent of) child externalizing behavior (Lansford et al., 2011; Lengua, 2006), the consensus seems to be that it causes and worsens behavioral problems in children rather than the converse. However, it also appears that its adverse effects are not uniform, tending to vary by ethnicity and cultural norms (Lansford et al., 2005; Lansford, Deater-Deckard, Dodge, Bates, & Pettit, 2004), parental sensitivity and warmth (Deater-Deckard, Ivy, & Petrill, 2006), and child temperamental or self-regulatory characteristics (Erath, El-Sheikh, & Mark Cummings, 2009). HPD and environmental adversity are interrelated (Sturge-Apple, Suor, & Skibo, 2014), although not consistently (Kiernan & Huerta, 2008), and they are frequently examined jointly to predict child problem behavior. However, even studies showing that these two factors work together to predict child problem behavior do not test if they are causally or interactively linked. Some research shows that environmental adversity moderates (amplifies in this case) the effect of HPD on child adjustment and behavior (Callahan, Scaramella, Laird, & Sohr-Preston, 2011; MacKenzie, Nicklas, Brooks-Gunn, & Waldfogel, 2014). However, other research suggests that HPD mediates the effect of environmental adversity (Krishnakumar, Narine, Roopnarine, & Logie, 2014; Lansford et al., 2004; Pinderhughes, Dodge, Bates, Pettit, & Zelli, 2000; Rijlaarsdam et al., 2013), in line with the family stress model (Conger & Conger, 2002; Conger, Conger, & Martin, 2010). The family stress model predicts that challenges associated with financial difficulties in the family and adverse life events (ALE) affect parental psychological functioning, which, in turn, influences child emotional and behavioral outcomes through its effect on parenting.

However, HPD may also act as a moderator of the effect of environmental adversity on child behavior, such that children growing up in poor or stress-ridden households may be especially vulnerable to behavioral and emotional difficulties if they also experience harsh parenting. In other words, children experiencing the cumulative effects of environmental adversity and HPD may be most at risk. Although to our knowledge no study has examined this research question, there is, as discussed, some evidence from small-scale (Callahan et al., 2011) or cross-sectional (MacKenzie et al., 2014) studies showing that the two interact. To be sure, there is much research showing that other aspects of parenting can moderate the effects of environmental adversity on child behavior (Gunnar & Quevedo, 2007). For example, nurturing parenting, particularly warmth and sensitivity, as well as parental involvement in play and daily care, can favorably mold the stress-response tendencies of children exposed to high levels of environmental risk (Burchinal, Roberts, Zeisel, & Rowley, 2008; Dearing, 2004; Kim-Cohen, Moffitt, Caspi, & Taylor, 2004; Malmberg & Flouri, 2011; Masten & Shaffer, 2006). Elucidating how HPD may be related to the longitudinal association between environmental adversity and problem behavior in young children is important because it will show whether, for example, targeting harsh parenting for all or the most disadvantaged or stressed families may be the most effective prevention and intervention approach to reducing levels of early behavioral and emotional difficulties in children. The present study was performed to do this.

## The Present Study

We analyzed data from the U.K.’s Millennium Cohort Study (MCS), a large cohort of families with young children, followed longitudinally from age 9 months. We explored the longitudinal associations among environmental adversity (family SED and ALE), HPD, and child problem behavior (i.e., conduct, hyperactivity, and emotional problems) across the early- and middle-childhood data sweeps in MCS, corresponding to ages 3, 5, and 7 years. We also tested if HPD mediates or moderates the effect of environmental adversity on children’s trajectories of problem behavior. Our research questions were as follows:

1 Is there concurrently and longitudinally a relation between environmental adversity and child problem behavior?
2 Does HPD mediate this relation?
3 Does HPD moderate this relation?

We hypothesized that children exposed to environmental adversity would concurrently and longitudinally have more problem behavior relative to children without this exposure. We further hypothesized that HPD would partially mediate the effect of environmental adversity on child problem behavior, but also moderate it. Finally, we expected that HPD would be strongly associated with child problem behavior, given that, similar to environmental adversity, it is a powerful risk factor of child problem behavior in its own right.

## Method

### Participants and Procedure

MCS (http://wws.cls.ioe.ac.uk/mcs) is a longitudinal survey drawing its sample from all births in the United Kingdom over a year, from September 1, 2000. The sample was disproportionately stratified to ensure adequate numbers in the four U.K. countries and electoral wards with disadvantaged and, in England, ethnic minority populations (Plewis, 2007). Ethical approval for the MCS was gained from National Health Survey Multi-Centre Ethics Committees, and parents gave informed consent before interviews took place. Sweep 1–4 took place when the children were around 9 months and 3, 5, and 7 years. All main variables (child problem behavior, HPD, and environmental adversity) were measured in Sweeps 2–4. We used records for only one child per family (the firstborn when there were twins or triplets). Our analytic sample comprised children with a score for problem behavior in at least one of Sweeps 2–4 (*n* = 16,916), the majority of the MCS families (*n* = 19,244). Complete data on child problem behavior were not necessary because growth curve modeling, which we adopted, is able to handle unbalanced data.

### Measures

Emotional, conduct, and hyperactivity problems were measured with the main parent-reported Strengths and Difficulties Questionnaire (SDQ; Goodman, 1997). Each of the three scales has five items, scored 0 to 2 (*not true*, *somewhat true*, and *certainly true*, respectively). Sample items include “many worries, often seems worried” (emotional symptoms), “often has temper tantrums or hot tempers” (conduct problems), and “restless, overactive, cannot stay still for long” (hyperactivity). In our sample, internal consistency was at acceptable levels and in line with other SDQ research (Stone, Otten, Engels, Vermulst, & Janssens, 2010). Cronbach’s α values across the three sweeps ranged from .51 to .65 for emotional symptoms, .56 to .68 for conduct problems, and .71 to .79 for hyperactivity.

Environmental adversity was operationalized as family SED (poverty) and the number of potential ALE the family experienced between sweeps. SED was measured as the sum of four binary indicators (as in Malmberg & Flouri, 2011) of the family’s level of economic deprivation. This measure captures poverty and its associated material conditions more broadly than relying on measured income alone, and it emphasizes the interrelations between family-level socioeconomic risk factors. The four items are overcrowding (>1.5 people per room excluding bathroom and kitchen), not owning the home, receipt of means-tested income support, and income poverty (below the poverty line, set for equivalized net family income at 60% of the U.K. national median household income). ALE were measured as the number (out of 11) of potentially stressful life events experienced by the family between two consecutive sweeps. The events, derived from available MCS data and based on Tiet et al.’s (1998) Adverse Life Events Scale, are family member died, negative change in financial situation, new stepparent, sibling left home, child got seriously sick or injured, divorce or separation, family moved, parent lost job, new natural sibling, new stepsibling, and maternal depression (treated for or diagnosed with depression). At each sweep, the number of events occurring since the previous sweep was summed to form a total ALE score.

HPD was assessed in MCS with three items (on 5-point scales: *never*, *rarely*, *once a month*, *once a week*, *daily*) from the Conflict Tactics Scale (Straus & Hamby, 1997). The items measure how often the parent uses the following physical and verbal discipline tactics when the child misbehaves: smacks, shouts, and “tells off.” A total score was generated by summing the responses so that higher values indicated more frequent use of these tactics (α = .66 to .67 across sweeps).

We adjusted for selected child and family or parent characteristics to rule out confounders of the relation between adversity and child adjustment. Key covariates were gender, ethnicity, maternal education, and family structure. We also adjusted for temperament, recognizing the importance of child effects on harsh parenting (Lengua, 2006). In MCS temperament was assessed with a summary score of 14 items of the Carey Infant Temperament Scale (Carey & McDevitt, 1978) at age 9 months. These items index three dimensions of the baby’s temperament: mood, adaptability, and regularity or rhythmicity. A higher score on the scale indicated an easier temperament (α = .64). With regard to the other child-level covariates, girls, in general, are at lower risk of behavioral problems than boys (Egger & Angold, 2006). The main ethnic minority groups in the United Kingdom have similar or lower rates of emotional, behavioral, and hyperactivity problems than White British children (Goodman, Patel, & Leon, 2008), despite experiencing more poverty (Platt, 2007). With regard to the family-level covariates, family structure (two parents or not) was time-varying, measured at ages 3, 5, and 7 years. Maternal education was measured as the mother’s highest academic qualification achieved by the end of our study period (age 7 years), coded as university degree or not. Maternal education and family structure are strongly related to family risk and child problem behavior (Evans & English, 2002; Kiernan & Huerta, 2008).

### Analytic Strategy

First, we investigated whether the families in our analytic sample (*n* = 16,916) were different (at *p* < .05) from those not in it (*n* = 2,328) on our study variables. We then explored levels and patterns of missingness in our covariates to decide on our approach to dealing with missing data. After this, we inspected the correlations between our main variables. Finally, we explored the shape of children’s average trajectory of each outcome and fitted two-level growth curve models (Snijders & Bosker, 1999) in which occasions of SDQ measurements (Level 1) were nested in children (Level 2). Growth curve models allowed us to estimate the average level of problems at a particular time point and the average growth rate in problems over time. By specifying a random linear slope on the child’s age to allow for changes in problems across time to vary between children, we could also model individual trajectories of problems from ages 3 to 7 years. We fitted fixed and random linear slopes, and we included a fixed quadratic term to account for the curved shape of children’s average trajectories (see next paragraph). The stratified sampling design of MCS was recognized by including the nine MCS strata in all models: England-advantaged, England-disadvantaged, England-ethnic, Wales-advantaged, Wales-disadvantaged, Scotland-advantaged, Scotland-disadvantaged, Northern Ireland-advantaged, and Northern Ireland-disadvantaged. These are subgroups of the population from which cohort families were sampled. As explained, cohort families were oversampled from disadvantaged areas, areas with high proportions of ethnic minorities in England, and the three smaller U.K. countries.

The full sequence of models estimated is outlined in Table 1. Model 1 included age (grand mean centered at age 5.22 years) and its square (because the average trajectories for all three problem types were curvilinear). Grand mean centering age at the midpoint minimizes the correlation between age and age-squared, thus stabilizing the estimation (Raudenbush & Bryk, 2002). Also included were the MCS design strata and environmental adversity (SED and ALE), as well as interaction terms for environmental adversity and age and for environmental adversity and age-squared. This model enabled us to examine whether the level of problems at around age 5 and the rate of change in problems over time shifted with SED and ALE. Model 2 included HPD, also specified as a main and an interactive (with age and age-squared) effect. Therefore, this model tested whether HPD mediates the effect of environmental adversity. Model 3, adding the child and family covariates, tested the robustness of all effects identified. Models 4 and 5 were estimated to test the role of HPD in moderating the effects of environmental adversity on child problem behavior. These models separately investigated the interactions between SED and HPD and between ALE and HPD at central age and on the trajectories.[Table-anchor tbl1]

## Results

### Bias Analysis and Descriptives

Differences between the analytic and the nonanalytic samples were small (Tables 2 and 3). In the former, there was a slight overrepresentation and underrepresentation, respectively, of Black and “other” ethnic children, and a greater proportion of two-parent families at age 3. The analytic sample also experienced fewer stressful life events, but only at the beginning of the study period (age 3), as well as less poverty throughout. On average, the analytic sample had less than one element of poverty and experienced one to two stressful life events at each age. With regard to HPD, the average score of 8.36 for age 5, for example, indicates that the average parent used at least two of the three tactics once a month when their child was around age 5.[Table-anchor tbl2][Table-anchor tbl3]

Environmental adversity and HPD were significantly correlated with the three types of child problems across all sweeps (see Table 4). HPD had a weak relation with ALE, and an even weaker (ranging 0.02 to 0.05) but negative relation with SED, such that more frequent use of HPD was related to lower levels of poverty. As expected, SED was very stable during the study period, but all other main variables showed only moderate stability.[Table-anchor tbl4]

### Missing Data Analysis and Imputation

Because of some missingness in our study variables (7–10% of values were missing across sweeps), we multiply imputed missing data on the covariates. We generated five imputed data sets (Allison, 2009; Graham, Olchowski, & Gilreath, 2007) in SPSS20 using the Markov chain Monte Carlo procedure. In the imputation model we included all covariates as predictor and predicted variables. We fitted our models in Stata13 using the multiple imputation estimate command, which performs individual analyses for each of the imputed data sets, collects estimates of coefficients and their variance covariance estimates, and reports the pooled results.

### Growth Curve Models

In Model 1 (see Table 1 in the online supplementary material), SED and ALE were associated with conduct, hyperactivity, and emotional problems at around age 5. SED was also related to the linear rate of change in conduct problems and to the change in the linear rate of change in conduct problems (i.e., the linear and the quadratic term, respectively, were significant), but it was unrelated to the linear rate of change over time in hyperactivity and emotional symptoms. ALE predicted the linear rate of change only in emotional symptoms. In Model 2 (see Table 2 in the online supplementary material), the effects of HPD were significant on all three problem types at age 5. HPD was also related to the linear rate of change in hyperactivity and emotional problems. The effects of SED and ALE remained significant; therefore, they were not fully explained by HPD. However, HPD may still be a partial mediator of these effects. (Although possible, this seems unlikely given the weak correlation of environmental adversity with HPD, as discussed in the previous paragraph and shown in Table 4.) We were unable to test this in Stata using multiply imputed data.

Adding the child and family covariates in Model 3 (see Table 5) did not attenuate the effects (main or interactive with age and age-squared) of either environmental adversity or harsh parenting. This model also showed that girls had significantly fewer conduct and hyperactivity problems, but more emotional symptoms, and that there were several ethnic differences in child adjustment. In particular, Black (relative to White) children had fewer conduct, hyperactivity, and emotional problems. Pakistani and Bangladeshi children were more hyperactive and had more emotional symptoms than White children, and children from other ethnic groups had more emotional problems. Easy temperament was negatively associated with conduct, hyperactivity, and emotional problems, as was living in an intact family and with a university-educated mother.[Table-anchor tbl5]

Models 4 and 5 (see Tables 3 and 4 in the online supplementary materials) showed evidence for several moderator effects of HPD. The effect of SED on conduct problems at central age was moderated by HPD (*b* = 0.014, *SE* = 0.005, *p* < .01, *r* = .072^1^). Furthermore, harsh parenting moderated the effect of SED on the linear rate of change in conduct problems over time (*b* = 0.005, *SE* = 0.002, *p* < .01, *r* = .103). HPD also moderated several ALE effects. As with SED, harsh parenting interacted with ALE to predict the level of conduct problems at central age (*b* = 0.012, *SE* = 0.005, *p* < .05, *r* = .013) and the linear rate of their change (*b* = 0.006, *SE* = 0.002, *p* < .01, *r* = .060). The same was found for emotional symptoms, such that harsh parenting concurrently (*b* = 0.010, *SE* = 0.003, *p* < .01, *r* = .008) and longitudinally (*b* = 0.010, *SE* = 0.002, *p* < .01, *r* = .112) moderated the effect of ALE. Furthermore, the effect of SED on emotional symptoms at central age was moderated by HPD (*b* = 0.008, *SE* = 0.003, *p* < .05, *r* = .093). For hyperactivity, the effect of ALE at around age 5 was moderated by harsh parenting (*b* = 0.013, *SE* = 0.006, *p* < .05, *r* = .025).

**Figure 1 fig1:**
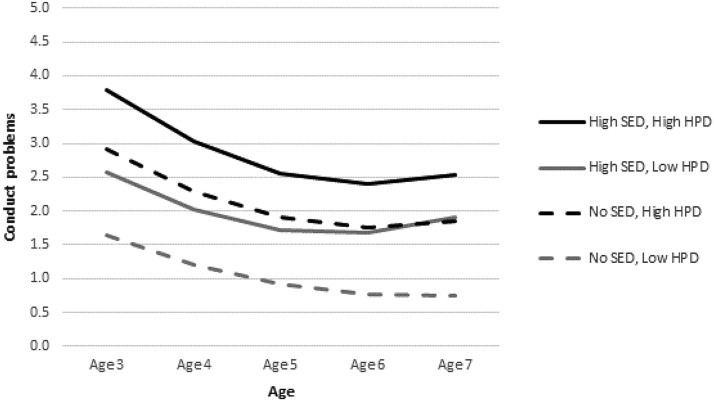
Predicted conduct problem trajectories for children by high/low HPD and high/no SED (Model 4). The predictions are plotted for the reference group for each categorical variable, except for family structure, and at the mean of each continuous variable.

**Figure 2 fig2:**
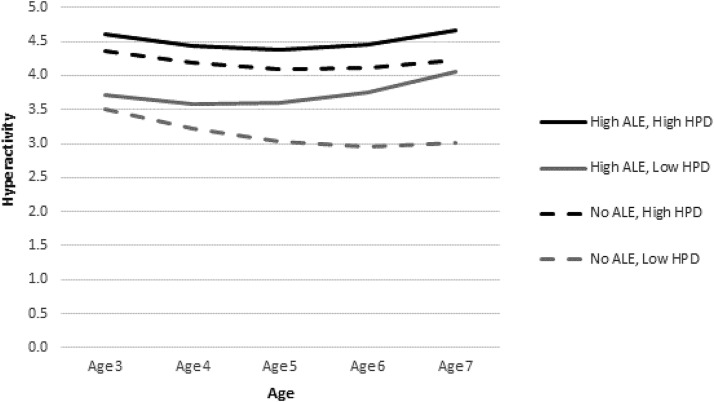
Predicted hyperactivity trajectories for children by high/low HPD and high/no ALE (Model 5). See note for Figure 1.

**Figure 3 fig3:**
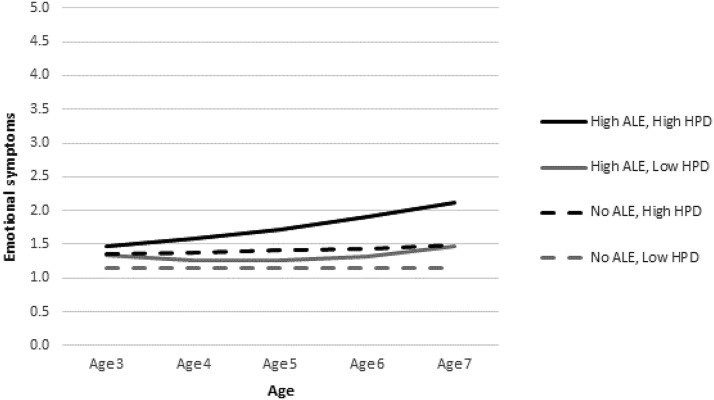
Predicted emotional symptom trajectories for children by high/low HPD and high/no ALE (Model 5). See note for Figures 1–2.

To unpack the interactions between environmental adversity and harsh parenting, we plotted the predicted trajectories of problems for illustrative cases with high and low levels of adversity by high and low levels of harsh parenting (Figures 1–3). Low and high levels of adversity corresponded to the 10th (i.e., zero elements of SED or ALE) and 90th (i.e., three elements of SED or ALE) percentiles, respectively. A high level of harsh parenting was defined by a HPD score at the 90th percentile. A low level of harsh parenting corresponded to a score at the 10th percentile. We present three figures (one based on Model 4 and two based on Model 5 results) to demonstrate graphically how harsh parenting might moderate the effects of environmental adversity on child problem behavior.[Fig-anchor fig1][Fig-anchor fig2][Fig-anchor fig3]

Starting with conduct problems, Figure 1 displays the significant interaction between SED and HPD at age 5 and on the linear rate of change in problems. As can be seen, harsh parenting interacts with poverty to predict conduct problems. Children with SED and high HPD have the highest conduct problem scores. However, HPD also seems to affect children in the low-risk group. In fact, there is a somewhat larger gap between the two predicted trajectories (for low and high HPD) for children without SED than between those for their high-SED counterparts. Furthermore, this gap widens between ages 6 and 7 such that the no-SED child increases in conduct problems if she experiences high HPD whereas the no-SED child with low HPD continues to drop further in the level of problems. Turning to hyperactivity (see Figure 2), we see a similar interactive relation of ALE and HPD. Again, there is a larger gap between no-ALE children with high and low HPD than between high-ALE children with high and low HPD. On the other hand, the interaction between ALE and HPD on emotional symptoms (see Figure 3) demonstrates that HPD exacerbates the negative effect of ALE, but it does not differentiate substantially the trajectories of the no-ALE children. The child with high ALE and high HPD increases steadily in emotional symptoms across the study period. The child with high ALE and low HPD increases only slightly across the study period, maintaining a relatively flat trajectory. There is a small gap between children with no ALE who experience high and low HPD, with their trajectories staying roughly parallel and flat over time.

## Discussion

HPD has attracted the interest of developmental psychologists because it is strongly related to (levels of and changes in) child problem behavior but also to important risk factors of child problem behavior, such as poverty and ALE. However, previous research has not shown if harsh parenting explains or moderates the effect of such environmental adversity on problem behavior in young children. Using longitudinal data from a large, nationally representative U.K. cohort of children followed from preschool age to middle childhood, we performed this study to answer this question. Our study showed that, as expected, environmental adversity was related to internalizing (emotional) and externalizing (conduct and hyperactivity) problems in children. Also aligned with previous research (Gershoff, 2002) was our finding that harsh parenting was a risk factor of, particularly, externalizing problems in children. However, HPD did not explain, as we expected, why the children of poor and stressed families had more externalizing and internalizing difficulties. In fact, in this study, HPD was very weakly related to environmental adversity. Nonetheless, HPD moderated the effect of environmental adversity on internalizing and externalizing difficulties in children. It increased the level of internalizing and externalizing problems in children exposed to high levels of adversity, but it also differentiated the level of externalizing problems in children exposed to low levels of environmental adversity and was associated with an increase in internalizing problems in these children over time.

The finding that harsh parenting and environmental adversity, especially family poverty, were weakly interrelated runs counter to much research showing that harsh parenting tends to be socioeconomically patterned. Other research using MCS, the data set that we used in this study, has also shown that socioeconomic deprivation and harsh or ineffective parenting, at least when parent reported, are not interrelated, although socioeconomic deprivation and warm parenting are (Flouri, Midouhas, Joshi, & Tzavidis, 2015; Kiernan & Huerta, 2008). Warmth and harshness are orthogonal parenting dimensions, as other research has shown (Deater-Deckard et al., 2006); therefore, they can be associated differently with family socioeconomic risk.

It was also somewhat surprising to find that, for conduct problems and hyperactivity, HPD appeared to have a greater effect on children from low rather than high-risk families. Perhaps children’s externalizing problems may be influenced more by poverty and other adversities, which tend to be more ongoing, with discipline tactics having less of an additive effect when simultaneously occurring. With regard to emotional symptoms, we see clearly that HPD was a vulnerability factor for children with high levels of adversity. Nevertheless, the children with the most serious problems, in general, were those who experience high levels of environmental adversity and high levels of HPD. Therefore, targeting poor and harsh-disciplining families may be a more effective prevention and intervention strategy to reduce levels of child problem behavior than targeting families classified as high risk on the basis of either one of these independent risk factors. However, in contrast to SED, HPD varied more over time, suggesting that it is probably a response to external stimuli or changes rather than a manifestation of the parents’ “character,” for example, as reflected in their values or personality traits. This confirms previous findings showing that harsh parenting is a response to as well as a determinant of externalizing problems in children (Lansford et al., 2011; Lengua, 2006). In this study we did not explore such bidirectionality in depth, but we adjusted for child temperament; therefore, we are confident of the robustness of our findings. We note that our findings were also robust to adjustment for parental warmth (results available on request), suggesting that the effect of HPD on increasing child problem behavior and moderating the effect of environmental adversity on child problem behavior was not attenuated by parental warmth. Nonetheless, future studies should also explore how parental warmth, HPD, and environmental adversity may interact. As much research has shown, it is important to examine the effects of harsh parenting in the broader context in which it occurs (Mendez, Durtschi, Neppl, & Stith, 2016).

The findings about this robustness of the adverse effect of HPD and about the lack of an association between HPD and SED run counter to much research, especially with samples in the United States. Whether this variation reflects differences in cultural context or methodology (e.g., in the type or content of measurement instruments) is unclear. Cross-country (and therefore, to an extent, cross-cultural) research using similar methodology will answer this question. Our study has some additional limitations. First, as a correlational study it is unable to prove that greater exposure to HPD caused children to be more vulnerable to risk, or that little use of HPD led them to be resilient to risk. Second, some of these convergences could have been produced by regression to the mean, in which extremely high (and low) intercept and slope values affected by measurement error are likely to be closer to the sample mean at repeat assessments. Third, with only three time points of data on emotional and behavioral problems, the possibilities for modeling the functional form of children’s individual trajectories were limited. Fourth, the reliance on parental (usually maternal) reports to measure children’s emotional and behavioral problems and HPD means that correlations between these measures are likely inflated by the idiosyncrasies of the reporter. Related to this, HPD reports might be subject to biases related to social desirability. However, in MCS, HPD was only measured by parent report. As for emotional and behavioral problems, in the MCS sweeps only the main parent (the mother in the vast majority of cases) was asked to complete the SDQ. MCS has some teacher-reported SDQ data available, but only for England and Wales and only for age 7 years. Last, our measures of emotional and conduct problems at age 3 years and our measure of HPD had fairly low reliability, which suggests that findings should be interpreted with caution. Despite these limitations, our study showed that, among young U.K. families, HPD increases children’s problem behavior and moderates the effects of family poverty and family upheaval on children’s problem behavior. Research using data from the next sweeps of MCS, when they become available, will show if these main and moderator effects of harsh parenting persist as extrafamilial settings become more important for children.

## Supplementary Material

10.1037/fam0000258.supp

## Figures and Tables

**Table 1 tbl1:** Model Specification

Model	Specification
1	Age + Age^2^ + SED + SED × Age + SED × Age^2^ + ALE + ALE × Age + ALE × Age^2^ + MCS Design Strata^a^
2	Model 1 + HPD + HPD × Age + HPD × Age^2^
3	Model 2 + Child^b^ + Parent^c^ Covariates
4	Model 3 + SED × HPD + SED × HPD × Age + SED × HPD × Age^2^
5	Model 4 + ALE × HPD + ALE × HPD × Age + ALE × HPD × Age^2^
*Note.* SED = socioeconomic disadvantage; ALE = adverse life events; HPD = Harsh parental discipline; MCS = Millennium Cohort Study.	
^a^ England-advantaged (reference group), England-disadvantaged, England-ethnic, Wales-advantaged, Wales-disadvantaged, Scotland-advantaged, Scotland-disadvantaged, Northern Ireland-advantaged, and Northern-Ireland-disadvantaged. ^b^ Gender; ethnicity; temperament. ^c^ Mother is university educated or not; two-parent family or not.	

**Table 2 tbl2:** Descriptives of Categorical Study Variables in the Analytic and Nonanalytic Samples

	Analytic sample (*n* = 16,916)		Nonanalytic sample (*n* = 2,328)		Test *F*
Variable	*n*	%	*n*	%	
Child					
Girl	8,288	48.13	1,061	46.03	1.59
Ethnicity					3.15**
White	14,062	75.76	1,679	77.30	
Black	596	7.03	133	5.14	
Indian	430	2.65	67	2.57	
Pakistani or Bangladeshi	1,077	8.98	273	8.61	
Mixed	512	4.15	82	3.62	
Other	230	1.43	73	2.76	
Parent or household					
Mother is university educated	2,821	10.71	205	9.34	2.85
Two-parent family					
Sweep 2	12,195	79.73	139	65.26	11.02***
Sweep 3	13,237	74.62	103	73.37	0.06
Sweep 4	9,920	69.30	72	78.65	2.70
*Note*. *F* = *F* statistic for design-based Pearson χ^2^ (converted to *F* test to account for the MCS sampling design). Proportions are weighted to account for sampling design and nonresponse in MCS. *n* values are unweighted.					
** *p* < .01. *** *p* < .001.					

**Table 3 tbl3:** Descriptives of Continuous Time-Varying Study Variables in the Analytic and Nonanalytic Samples

	Analytic sample (*n* = 16,916)				Nonanalytic sample (*n* = 2,328)			
Variable	*n*	*M* (*SE*)	Range	95% CI	*n*	*M* (*SE*)	Range	95% CI
Age (years)								
Age 3	15,369	3.14 (0.00)	2.65–4.57	[3.13, 3.14]	212	3.22 (0.03)	3.00–4.38	[3.17, 3.28]
Age 5	15,102	5.21 (0.00)	4.41–6.13	[5.20, 5.22]	142	5.17 (0.03)	4.44–5.81	[5.11, 5.23]
Age 7	13,765	7.24 (0.00)	6.34–8.15	[7.16, 7.30]	92	7.23 (0.01)	6.71–7.82	[7.22, 7.24]
SED								
Age 3	12,909	0.84 (0.03)	0–4	[0.79, 0.87]	94	2.06 (0.17)	0–4	[1.73, 2.38]
Age 5	12,806	0.86 (0.02)	0–4	[0.81, 0.90]	60	2.15 (0.16)	0–4	[1.83, 2.47]
Age 7	13,607	0.86 (0.03)	0–4	[0.81, 0.91]	75	1.76 (0.15)	0–4	[1.47, 2.06]
ALE								
Age 3	16,916	1.61 (0.02)	0–7	[1.57, 1.64]	2328	1.12 (0.09)	0–5	[0.94, 1.31]
Age 5	16,916	1.45 (0.03)	0–7	[1.43, 1.48]	2328	1.48 (0.11)	0–5	[1.26, 1.69]
Age 7	16,916	1.45 (0.01)	0–6	[1.42, 1.48]	2328	1.56 (0.09)	0–3	[1.38, 1.75]
HPD								
Age 3	12,988	9.38 (0.03)	3–15	[9.32, 9.44]	9	8.46 (1.40)	3–14	[5.70, 11.21]
Age 5	14,138	8.36 (0.02)	3–15	[8.31, 8.40]	6	8.92 (1.46)	6–14	[6.05, 11.78]
Age 7	12,446	8.11 (0.02)	3–15	[8.06, 8.15]	1	8 (−)*	—	—
*Note*. SED = socioeconomic disadvantage; ALE = adverse life events; HPD = Harsh parental discipline. All means are weighted to account for sampling design and nonresponse in MCS. *n* values are unweighted. *SE*s and CIs are adjusted for clustered sampling except in cases marked * based on a sample represented by a single primary sampling unit (i.e., ward) for a given sampling stratum. These discrepancies arise occasionally because of migration out of the original strata. CI = confidence interval.								

**Table 4 tbl4:** Correlations Among the Main Variables in the Analytic Sample

	SED3y	SED5y	SED7y	ALE3y	ALE5y	ALE7y	Cond3y	Cond5y	Cond7y	Hyp3y	Hyp5y	Hyp7y	Emot3y	Emot5y	Emot7y	HPD3y	HPD5y	HPD7y
SED3y	1																	
SED5y	.84***	1																
SED7y	.78***	.83***	1															
ALE3y	.23***	.12***	.12***	1														
ALE5y	.10***	.21***	.16***	.13***	1													
ALE7y	.05***	.08***	.20***	.13***	.13***	1												
Cond3y	.26***	.24***	.24***	.15***	.08***	.07***	1											
Cond5y	.25***	.25***	.25***	.08***	.13***	.08***	.50***	1										
Cond7y	.24***	.24***	.24***	.09***	.12***	.16***	.44***	.59***	1									
Hyp3y	.21***	.20***	.20***	.13***	.07***	.06***	.49***	.47***	.34***	1								
Hyp5y	.22***	.21***	.22***	.08***	.12***	.08***	.38***	.53***	.43***	.57***	1							
Hyp7y	.20***	.20***	.19***	.08***	.09***	.13***	.35***	.44***	.54***	.51***	.68***	1						
Emot3y	.20***	.19***	.19***	.10***	.06***	.04***	.35***	.21***	.18***	.25***	.17***	.16***	1					
Emot5y	.16***	.16***	.17***	.07***	.11***	.06***	.24***	.32***	.23***	.19***	.27***	.20***	.42***	1				
Emot7y	.16***	.18***	.17***	.08***	.12***	.15***	.24***	.27***	.37***	.20***	.25***	.31***	.35***	.51***	1			
HPD3y	−.04***	−.03**	−.02	.09***	.05***	.06**	.34***	.22***	.21***	.24***	.21***	.19***	.06***	.06***	.07***	1		
HPD5y	−.04***	−.05***	−.04***	.07***	.05***	.06***	.24***	.33***	.26***	.19***	.26***	.23***	.03***	.10***	.10***	.56***	1	
HPD7y	−.03**	−.03**	−.03**	.06***	.06***	.09***	.24***	.28***	.36***	.19***	.25***	.30***	.04***	.07***	.12***	.51***	.62***	1
*Note.* 3, 5, and 7 refer to age in years. SED = socioeconomic disadvantage; ALE = adverse life events; HPD = Harsh parental discipline; Cond = Conduct (problems), Hyp = Hyperactivity; Emot = Emotional (symptoms).																		
** *p* < .01. *** *p* < .001.																		

**Table 5 tbl5:** Fixed Effects Estimates and Variance Covariance Estimates for Model 3 Predicting Conduct Problems, Hyperactivity, and Emotional Symptoms

	Conduct problems (*n* = 16,908)			Hyperactivity (*n* = 16,884)			Emotional symptoms (*n* = 16,908)		
Variables	Coeff.	*SE*	95% CI	Coeff.	*SE*	95% CI	Coeff.	*SE*	95% CI
Fixed effects									
Constant	1.248***	0.106	[1.041, 1.455]	3.390***	0.164	[3.069, 3.712]	2.545***	0.108	[2.334, 2.757]
Age	−0.215***	0.018	[−0.251, −0.180]	−0.291***	0.024	[−0.338, −0.244]	−0.114***	0.018	[−0.150, −0.078]
Age^2^	0.011***	0.012	[−0.013, 0.036]	0.075***	0.017	[0.043, 0.108]	0.016	0.013	[−0.010, −0.041]
Girl	−0.218***	0.019	[−0.218, 0.019]	−0.634***	0.029	[−0.691, −0.576]	0.043*	0.019	[0.005, 0.081]
Ethnicity (Ref: White)									
Mixed	−0.082	0.057	[−0.194, 0.030]	−0.020	0.088	[−0.193, 0.153]	−0.066	0.058	[−0.181, 0.043]
Indian	−0.041	0.067	[−0.173, 0.091]	0.156	0.104	[−0.048, 0.360]	0.065	0.069	[−0.071, 0.200]
Pakistani or Bangladeshi	−0.067	0.051	[−0.168, 0.033]	0.307***	0.080	[0.151, 0.463]	0.485***	0.053	[0.382, 0.588]
Black	−0.556***	0.059	[−0.671, −0.441]	−0.515***	0.091	[−0.693, −0.338]	−0.281***	0.060	[−0.399, −0.163]
Other	−0.134	0.089	[−0.308, 0.041]	0.064	0.136	[−0.203, 0.331]	0.350***	0.090	[0.174, 0.527]
(Easy) temperament	−0.026***	0.002	[−0.029, −0.023]	−0.634***	0.029	[−0.033, −0.024]	−0.035***	0.002	[−0.038, −0.032]
Mother is university educated	−0.327***	0.025	[−0.377, −0.276]	−0.760***	0.039	[−0.837, −0.683]	−0.176***	0.026	[−0.226, −0.125]
Two-parent family	−0.205***	0.023	[−0.250, −0.160]	−0.350***	0.034	[−0.416, −0.283]	−0.061*	0.024	[−0.108, −0.014]
SED	0.183***	0.011	[0.161, 0.206]	0.177***	0.016	[0.146, 0.209]	0.110***	0.012	[0.087, 0.134]
SED*Age	−0.028***	0.004	[−0.035, −0.020]	−0.005	0.005	[−0.014, 0.005]	−0.002	0.004	[−0.009, 0.005]
SED*Age^2^	0.014***	0.002	[0.009, 0.019]	−0.005	0.003	[−0.011, 0.001]	−0.006*	0.003	[0.001, 0.011]
ALE	0.047***	0.010	[0.028, 0.066]	0.061***	0.013	[0.035, 0.087]	0.071***	0.010	[0.050, 0.091]
ALE*Age	−0.003	0.004	[−0.010, 0.005]	−0.0002	0.005	[−0.010, 0.010]	0.026***	0.004	[0.019, 0.034]
ALE*Age^2^	0.012***	0.003	[0.006, 0.017]	0.005	0.004	[−0.003, 0.012]	0.007*	0.003	[0.001, 0.013]
HPD	0.210***	0.005	[0.199, 0.221]	0.235***	0.008	[0.220, 0.250]	0.074***	0.006	[0.063, 0.085]
HPD*Age	−0.003	0.002	[−0.007, 0.001]	0.026***	0.003	[0.021, 0.031]	0.016***	0.002	[0.012, 0.020]
HPD*Age^2^	0.009***	0.001	[0.006, 0.011]	−0.002	0.002	[−0.006, 0.002]	−0.002	0.001	[−0.005, 0.001]
Area stratum (Ref: England-advantaged)									
England-disadvantaged	0.221***	0.027	[0.168, 0.274]	0.227***	0.042	[0.145, 0.309]	0.114***	0.027	[0.060, 0.168]
England-ethnic	0.121**	0.044	[0.034, 0.208]	0.158*	0.069	[0.024, 0.293]	0.116*	0.045	[0.027, 0.205]
Scotland-advantaged	−0.033	0.042	[−0.116, 0.050]	−0.208**	0.066	[−0.337, −0.080]	−0.056	0.043	[−0.141, 0.028]
Scotland-disadvantaged	0.105*	0.043	[0.022, 0.189]	0.108	0.066	[−0.022, 0.238]	−0.023	0.044	[−0.108, 0.063]
Northern Ireland-advantaged	−0.194***	0.051	[−0.295, −0.093]	−0.314***	0.080	[−0.470, −0.158]	−0.029	0.052	[−0.0131, 0.074]
Northern Ireland-disadvantaged	0.098*	0.043	[0.014, 0.182]	−0.052	0.066	[−0.182, 0.077]	0.053	0.044	[−0.032, 0.139]
Wales-advantaged	−0.006	0.048	[−0.100, 0.088]	−0.037	0.075	[−0.183, 0.109]	−0.082	0.049	[−0.178, 0.015]
Wales-disadvantaged	0.190***	0.035	[0.122, 0.259]	0.270***	0.054	[0.164, 0.377]	0.076*	0.036	[0.006, 0.146]
Random effects									
Level 2 (child)									
Between-child intercept variance	1.069***	0.018	[1.035, 1.105]	2.740***	0.041	[2.660, 2.822]	1.061***	0.019	[1.024, 1.099]
Between-child slope variance	0.090***	0.003	[0.084, 0.097]	0.118***	0.006	[0.108, 0.129]	0.053***	0.003	[0.047, 0.060]
Between-child intercept/slope variance covariance	−0.139***	0.005	—	0.097***	0.010	—	0.103***	0.005	—
Level 1 (occasion)									
Between-occasion variance	1.094***	0.014	[1.067, 1.122]	1.939***	0.025	[1.891, 1.987]	1.280***	0.016	[1.249, 1.312]
*Note*. *n* is not 16,916 because we did not impute missing values on the SDQ-dependent variables; Coeff. = coefficient; Ref = reference; SED = socioeconomic disadvantage; ALE = adverse life events; HPD = Harsh parental discipline.									
* *p* < .05. ** *p* < .01. *** *p* < .001.									

## Footnotes

1 This is the partial correlation, which serves as an effect size estimate. It can be easily computed from the *t* statistic, using the formula $r=\sqrt{\frac{t^{2}}{t^{2}+df}}$.
