# Supplementary material for: Environmental Adversity and Children’s Early Trajectories of Problem Behavior: The Role of Harsh Parental Discipline
Source: J Fam Psychol. 2016 Dec 15;31(2):234–43. doi: 10.1037/fam0000258 (PMC5327893; doi:10.1037/fam0000258)
Supplement: Supplementary file 1 [file ze7008163073so1.docx]

**Supplemental Materials**

**Environmental Adversity and Children's Early Trajectories of Problem Behaviour: The Role of Harsh Parental Discipline**

**by E. Flouri & E. Midouhas, 2016, *Journal of Family Psychology***

**http://dx.doi.org/10.1037/fam0000258**

**Supplementary Table 1**

*Fixed Effects Estimates and Variance Covariance Estimates for Model 1 Predicting Conduct Problems, Hyperactivity and Emotional Symptoms*

|  | **Conduct problems**  **(*n* = 16,908)** | | | **Hyperactivity**  **(*n* = 16,884)** | | | **Emotional symptoms**  **(*n* = 16,908)** | | | |
| --- | --- | --- | --- | --- | --- | --- | --- | --- | --- | --- |
|  | **Coeff.** | **SE** | **95% CI** | **Coeff.** | **SE** | **95% CI** | **Coeff.** | **SE** | | **95% CI** |
|  | **Fixed effects** | | | | | | | | | |
| Constant | 1.134*** | 0.026 | [1.083,1.185] | 2.854*** | 0.038 | [2.780,2.928] | 1.068*** | 0.026 | | [1.018,1.119] |
| Age | -0.315*** | 0.007 | [-0.329,-0.300] | -0.124*** | 0.010 | [-0.142,-0.105] | 0.004 | 0.007 | | [-0.150,-0.078] |
| Age^2^ | 0.097*** | 0.005 | [0.088,0.107] | 0.063*** | 0.007 | [0.051,0.076] | -0.001 | 0.005 | | [-0.011,-0.009] |
| SED | 0.226*** | 0.011 | [0.204,0.247] | 0.262*** | 0.015 | [0.233,0.292] | 0.148*** | 0.011 | | [0.126,0.169] |
| SED*age | -0.024*** | 0.004 | [-0.030,-0.015] | -0.005 | 0.005 | [-0.015,0.004] | -0.004 | 0.004 | | [-0.011,0.004] |
| SED*age^2^ | 0.014*** | 0.002 | [0.009,0.018] | -0.004 | 0.004 | [-0.011,0.003] | 0.006* | 0.003 | | [0.002,0.012] |
| ALE | 0.072*** | 0.010 | [0.052,0.091] | 0.093*** | 0.013 | [0.066,0.119] | 0.079*** | 0.010 | | [0.059,0.099] |
| ALE*age | -0.002 | 0.004 | [-0.010,0.006] | -0.004 | 0.005 | [-0.006,0.015] | 0.030*** | 0.004 | | [0.022,0.037] |
| ALE*age^2^ | 0.016*** | 0.003 | [0.011,0.022] | 0.007 | 0.004 | [-0.003,0.015] | 0.008** | 0.003 | | [0.002,0.013] |
| *Area stratum (Ref: England-advantaged)* |  |  |  |  |  |  |  |  | |  |
| England-disadvantaged | 0.266*** | 0.029 | [0.209,0.324] | 0.344*** | 0.044 | [0.257,0.430] | 0.174*** | 0.028 | | [0.119,0.228] |
| England-ethnic | 0.051 | 0.037 | [0.023,0.124] | 0.299*** | 0.057 | [0.188,0.409] | 0.419*** | 0.036 | | [0.348,0.449] |
| Scotland-advantaged | -0.035 | 0.047 | [-0.126,0.057] | -0.241** | 0.071 | [-0.379,-0.103] | -0.081 | 0.044 | | [-0.168,0.005] |
| Scotland-disadvantaged | 0.162* | 0.047 | [0.070,0.255] | 0.193** | 0.071 | [0.053,0.332] | 0.010 | 0.045 | | [-0.078,0.098] |
| Northern Ireland-advantaged | -0.159** | 0.057 | [-0.270,-0.048] | -0.320*** | 0.086 | [-0.488,-0.152] | -0.039 | 0.054 | | [-.0144,0.066] |
| Northern Ireland-disadvantaged | 0.148** | 0.047 | [0.056,0.240] | 0.021 | 0.071 | [-0.118,0.159] | 0.070 | 0.045 | | [-0.017,0.158] |
| Wales-advantaged | -0.025 | 0.053 | [-0.129,0.079] | -0.064 | 0.080 | [-0.221,0.094] | -0.108* | 0.050 | | [-0.206,0.009] |
| Wales-disadvantaged | 0.216*** | 0.039 | [0.140,0.291] | 0.346*** | 0.058 | [0.232,0.461] | 0.086* | 0.037 | | [0.014,0.158] |
|  | **Random effects** | | | | | | | |  |  |
| Level 2 (child) |  |  |  |  |  |  |  |  | |  |
| Between-child intercept variance | 1.390*** | 0.025 | [1.349,1.433] | 3.319*** | 0.047 | [3.228,3.412] | 1.144*** | 0.020 | | [1.106,1.184] |
| Between-child slope variance | 0.098*** | 0.004 | [0.091,0.105] | 0.126*** | 0.006 | [0.115,0.137] | 0.054*** | 0.003 | | [0.048,0.061] |
| Between-child intercept/slope variance covariance | -0.159*** | 0.006 | - | 0.130*** | 0.010 | - | 0.103*** | 0.005 | | - |
| Level 1 (occasion) |  |  |  |  |  |  |  |  | |  |
| Between-occasion variance | 1.132*** | 0.014 | [1.104,1.161] | 1.951*** | 0.025 | [1.903,2.000] | 1.287*** | 0.016 | | [1.255,1.319] |

*Note*: **p < .05, **p < .01, ***p < .001.*  *N* is not 16,916 because we did not impute missing values on the SDQ dependent variables. SED = Socio-economic disadvantage; ALE = Adverse life events.

**Supplementary Table 2**

*Fixed Effects Estimates and Variance Covariance Estimates for Model 2 Predicting Conduct Problems, Hyperactivity and Emotional Symptoms*

|  | **Conduct problems**  **(*n* = 16,908)** | | | **Hyperactivity**  **(*n* = 16,884)** | | | **Emotional symptoms**  **(*n* = 16,908)** | | | |
| --- | --- | --- | --- | --- | --- | --- | --- | --- | --- | --- |
|  | **Coeff.** | **SE** | **95% CI** | **Coeff.** | **SE** | **95% CI** | **Coeff.** | **SE** | | **95% CI** |
|  | **Fixed effects** | | | | | | | | | |
| Constant | -0.654*** | 0.051 | [-0.755,-0.554] | 0.830*** | 0.074 | [0.685,0.974] | 0.452*** | 0.054 | | [0.345,0.559] |
| Age | -0.215*** | 0.018 | [-0.251,-0.180] | -0.277*** | 0.024 | [-0.324,-0.230] | -0.109*** | 0.018 | | [-0.145,-0.073] |
| Age^2^ | 0.011*** | 0.012 | [-0.013,0.036] | 0.075*** | 0.017 | [0.043,0.108] | 0.015 | 0.013 | | [-0.011,-0.041] |
| SED | 0.248*** | 0.011 | [0.228,0.269] | 0.286*** | 0.015 | [0.257,0.315] | 0.154*** | 0.011 | | [0.132,0.175] |
| SED*age | -0.026*** | 0.004 | [-0.033,-0.019] | -0.004 | 0.005 | [-0.014,0.005] | -0.003 | 0.004 | | [-0.010,0.004] |
| SED*age^2^ | 0.014*** | 0.002 | [0.009,0.019] | -0.005 | 0.003 | [-0.011,0.002] | 0.006* | 0.003 | | [0.001,0.011] |
| ALE | 0.057*** | 0.010 | [0.039,0.077] | 0.078*** | 0.013 | [0.052,0.104] | 0.073*** | 0.010 | | [0.053,0.093] |
| ALE*age | -0.001 | 0.004 | [-0.009,0.007] | 0.002 | 0.005 | [-0.009,0.012] | 0.027*** | 0.004 | | [0.019,0.034] |
| ALE*age^2^ | 0.012*** | 0.003 | [0.006,0.017] | 0.004 | 0.004 | [-0.003,0.012] | 0.007* | 0.003 | | [0.001,0.013] |
| HPD | 0.214*** | 0.005 | [0.203,0.224] | 0.242*** | 0.008 | [0.226,0.257] | 0.074*** | 0.006 | | [0.063,0.085] |
| HPD*age | -0.003 | 0.002 | [-0.007,0.001] | 0.026*** | 0.003 | [0.020,0.031] | 0.015*** | 0.002 | | [0.011,0.019] |
| HPD*age^2^ | 0.009*** | 0.001 | [0.006,0.011] | -0.002 | 0.002 | [-0.006,0.002] | -0.002 | 0.001 | | [-0.005,0.001] |
| *Area stratum (Ref: England-advantaged)* |  |  |  |  |  |  |  |  | |  |
| England-disadvantaged | 0.227*** | 0.027 | [0.214,0.320] | 0.344*** | 0.042 | [0.260,0.427] | 0.175*** | 0.027 | | [0.120,0.229] |
| England-ethnic | 0.111** | 0.035 | [0.043,0.019] | 0.363*** | 0.054 | [0.256,0.469] | 0.436*** | 0.036 | | [0.366,0.507] |
| Scotland-advantaged | -0.057 | 0.043 | [-0.142,0.027] | -0.259*** | 0.068 | [-0.391,-0.126] | -0.087 | 0.044 | | [-0.174,-0.001] |
| Scotland-disadvantaged | 0.145* | 0.044 | [0.060,0.231] | 0.180** | 0.068 | [-0.047,0.314] | 0.006* | 0.045 | | [-0.082,0.093] |
| Northern Ireland-advantaged | -0.216*** | 0.052 | [-0.319,-0.114] | -0.368*** | 0.082 | [-0.528,-0.207] | -0.054 | 0.053 | | [-.159,0.051] |
| Northern Ireland-disadvantaged | 0.136* | 0.043 | [0.050,0.221] | 0.017 | 0.068 | [-0.116,0.151] | 0.069 | 0.044 | | [-0.018,0.156] |
| Wales-advantaged | -0.008 | 0.049 | [-0.104,0.088] | -0.043 | 0.077 | [-0.194,0.107] | -0.102* | 0.050 | | [-0.200,-0.004] |
| Wales-disadvantaged | 0.238*** | 0.036 | [0.168,0.307] | 0.370*** | 0.056 | [0.260,0.480] | 0.093* | 0.037 | | [0.213,0.164] |
|  | **Random effects** | | | | | | | |  |  |
| Level 2 (child) |  |  |  |  |  |  |  |  | |  |
| Between-child intercept variance | 1.129*** | 0.019 | [1.092,1.166] | 2.963*** | 0.044 | [2.879,3.050] | 1.125*** | 0.020 | | [1.087,1.164] |
| Between-child slope variance | 0.091*** | 0.003 | [0.084,0.097] | 0.118*** | 0.006 | [0.107,0.129] | 0.053*** | 0.003 | | [0.047,0.060] |
| Between-child intercept/slope variance covariance | -0.143*** | 0.005 | - | 0.102*** | 0.010 | - | 0.099*** | 0.005 | | - |
| Level 1 (occasion) |  |  |  |  |  |  |  |  | |  |
| Between-occasion variance | 1.100*** | 0.014 | [1.072,1.127] | 1.950*** | 0.025 | [1.902,2.000] | 1.282*** | 0.016 | | [1.251,1.315] |

*Note*: **p < .05, **p < .01, ***p < .001.*  *N* is not 16,916 because we did not impute missing values on the SDQ dependent variables. SED = Socio-economic disadvantage; ALE = Adverse life events; HPD = Harsh parental discipline.

**Supplementary Table 3**

*Fixed Effects Estimates and Variance Covariance Estimates for Model 4 Predicting Conduct Problems, Hyperactivity and Emotional Symptoms*

|  | **Conduct problems**  **(*n* = 16,908)** | | | **Hyperactivity**  **(*n* = 16,884)** | | | **Emotional symptoms**  **(*n* = 16,908)** | | | |
| --- | --- | --- | --- | --- | --- | --- | --- | --- | --- | --- |
|  | **Coeff.** | **SE** | **95% CI** | **Coeff.** | **SE** | **95% CI** | **Coeff.** | **SE** | | **95% CI** |
|  | **Fixed effects** | | | | | | | | | |
| Constant | 1.367*** | 0.114 | [1.143,1.590] | 3.380*** | 0.174 | [3.038,3.722] | 2.623*** | 0.116 | | [2.395,2.850] |
| Age | -0.176*** | 0.023 | [-0.221,-0.131] | -0.260*** | 0.029 | [-0.319,-0.202] | -0.088*** | 0.028 | | [-0.133,-0.044] |
| Age^2^ | -0.008*** | 0.016 | [-0.039,0.024] | 0.057** | 0.021 | [0.016,0.098] | 0.011 | 0.017 | | [-0.023,0.045] |
| Girl | -0.219*** | 0.019 | [-0.256,0.181] | -0.633*** | 0.029 | [-0.690,-0.575] | 0.042* | 0.019 | | [0.005,0.080] |
| *Ethnicity (Ref: White)* |  |  |  |  |  |  |  |  | |  |
| Mixed | -0.083 | 0.057 | [-0.195,0.029] | -0.019 | 0.088 | [-0.192,0.154] | -0.067 | 0.058 | | [-0.181,0.048] |
| Indian | -0.041 | 0.068 | [-0.173,0.092] | 0.159 | 0.104 | [-0.046,0.363] | 0.067 | 0.069 | | [-0.071,0.199] |
| Pakistani or Bangladeshi | -0.067 | 0.051 | [-0.168,0.034] | 0.307*** | 0.080 | [0.151,0.463] | 0.485*** | 0.053 | | [0.382,0.588] |
| Black | -0.554*** | 0.059 | [-0.669,-0.439] | -0.516*** | 0.091 | [-0.693,-0.338] | -0.280*** | 0.060 | | [-0.398,-0.161] |
| Other | -0.134 | 0.089 | [-0.308,0.040] | 0.064 | 0.136 | [-0.203,0.331] | 0.350*** | 0.090 | | [0.173,0.527] |
| (Easy) temperament | -0.026*** | 0.002 | [-0.029,-0.023] | -0.029*** | 0.002 | [-0.013,0.012] | -0.035*** | 0.002 | | [-0.038,-0.032] |
| Mother is university-educated | -0.327*** | 0.025 | [-0.377,-0.278] | -0.759*** | 0.039 | [-0.836,-0.682] | -0.176*** | 0.026 | | [-0.227,-0.125] |
| Two-parent family | -0.204*** | 0.023 | [-0.249,-0.159] | -0.351*** | 0.034 | [-0.417,-0.285] | -0.060* | 0.024 | | [-0.107,-0.013] |
| SED | 0.068*** | 0.040 | [-0.011,0.146] | 0.180** | 0.055 | [0.072,0.287] | 0.037 | 0.042 | | [-0.045,0.119] |
| SED*age | -0.070*** | 0.015 | [-0.101,-0.040] | -0.036 | 0.020 | [-0.076,0.004] | -0.030 | 0.016 | | [-0.060,0.001] |
| SED*age^2^ | 0.033*** | 0.012 | [0.010,0.056] | 0.015 | 0.015 | [-0.014,0.044] | -0.009 | 0.011 | | [-0.013,0.032] |
| ALE | 0.047*** | 0.010 | [0.028,0.066] | 0.061*** | 0.013 | [0.035,0.087] | 0.071*** | 0.010 | | [0.050,0.091] |
| ALE*age | -0.003 | 0.004 | [-0.010,0.005] | -0.0002 | 0.005 | [-0.011,0.010] | 0.026*** | 0.004 | | [0.019,0.034] |
| ALE*age^2^ | 0.012*** | 0.003 | [0.006,0.017] | 0.005 | 0.004 | [-0.003,0.012] | 0.007* | 0.003 | | [0.001,0.013] |
| HPD | 0.196*** | 0.007 | [0.182,0.210] | 0.236*** | 0.010 | [0.216,0.255] | 0.065*** | 0.008 | | [0.050,0.078] |
| HPD*age | -0.008** | 0.003 | [-0.013,0.003] | 0.023*** | 0.003 | [0.016,0.030] | 0.013*** | 0.003 | | [0.008,0.018] |
| HPD*age^2^ | 0.011*** | 0.002 | [0.008,0.014] | 0.001 | 0.002 | [-0.005,0.005] | -0.001 | 0.002 | | [0.008,0.018] |
| SED*HD | 0.014** | 0.005 | [0.005,0.008] | -0.0003 | 0.006 | [-0.013,0.012] | 0.009 | 0.005 | | [-0.006,0.018] |
| SED*HD*age | 0.005** | 0.002 | [0.002,0.008] | 0.003 | 0.002 | [-0.001,0.008] | 0.003 | 0.002 | | [-0.0001,0.007] |
| SED*HD*age^2^ | -0.002 | 0.001 | [-0.005,0.0004] | -0.002 | 0.002 | [-0.006,0.001] | -0.0004 | 0.001 | | [-0.003,0.002] |
| *Area stratum (Ref: England-advantaged)* |  |  |  |  |  |  |  |  | |  |
| England-disadvantaged | 0.222*** | 0.027 | [0.169,0.275] | 0.227*** | 0.042 | [0.145,0.308] | 0.115*** | 0.027 | | [0.061,0.169] |
| England-ethnic | 0.121** | 0.044 | [0.034,0.208] | 0.157* | 0.069 | [0.023,0.292] | 0.117* | 0.045 | | [0.028,0.206] |
| Scotland-advantaged | -0.032 | 0.042 | [-0.115,0.051] | -0.208** | 0.066 | [-0.337,-0.080] | -0.056 | 0.043 | | [-0.140,0.029] |
| Scotland-disadvantaged | 0.106* | 0.043 | [0.022,0.190] | 0.108 | 0.066 | [-0.021,0.238] | -0.023 | 0.044 | | [-0.108,0.063] |
| Northern Ireland-advantaged | -0.190*** | 0.051 | [-0.291,-0.090] | -0.316*** | 0.080 | [-0.472,-0.160] | -0.027 | 0.052 | | [-0.129,0.076] |
| Northern Ireland-disadvantaged | 0.097* | 0.043 | [0.014,0.181] | -0.052 | 0.066 | [-0.181,0.078] | 0.053 | 0.044 | | [-0.033,0.138] |
| Wales-advantaged | -0.007 | 0.048 | [-0.101,0.087] | -0.036 | 0.075 | [-0.182,0.110] | -0.082 | 0.049 | | [-0.178,0.014] |
| Wales-disadvantaged | 0.190*** | 0.035 | [0.122,0.259] | 0.270*** | 0.054 | [0.164,0.377] | 0.076* | 0.036 | | [0.006,0.146] |
|  | **Random effects** | | | | | | | |  |  |
| Level 2 (child) |  |  |  |  |  |  |  |  | |  |
| Between-child intercept variance | 1.069*** | 0.018 | [1.035,1.105] | 2.740*** | 0.041 | [2.660,2.821] | 1.060*** | 0.019 | | [1.023,1.098] |
| Between-child slope variance | 0.090*** | 0.003 | [0.084,0.097] | 0.118*** | 0.005 | [0.107,0.129] | 0.053*** | 0.003 | | [0.047,0.060] |
| Between-child intercept/slope variance covariance | -0.139*** | 0.005 | - | 0.097*** | 0.010 | - | 0.103*** | 0.005 | | - |
| Level 1 (occasion) |  |  |  |  |  |  |  |  | |  |
| Between-occasion variance | 1.094*** | 0.014 | [1.067,1.122] | 1.939*** | 0.025 | [1.891,1.988] | 1.280*** | 0.016 | | [1.249,1.313] |

*Note*: **p < .05, **p < .01, ***p < .001.*  *N* is not 16,916 because we did not impute missing values on the SDQ dependent variables. SED = Socio-economic disadvantage; ALE = Adverse life events; HPD = Harsh parental discipline.

**Supplementary Table 4**

*Fixed Effects Estimates and Variance Covariance Estimates for Model 5 Predicting Conduct Problems, Hyperactivity and Emotional Symptoms*

|  | **Conduct problems**  **(*n* = 16,908)** | | | **Hyperactivity**  **(*n* = 16,884)** | | | **Emotional symptoms**  **(*n* = 16,908)** | | | |
| --- | --- | --- | --- | --- | --- | --- | --- | --- | --- | --- |
|  | **Coeff.** | **SE** | **95% CI** | **Coeff.** | **SE** | **95% CI** | **Coeff.** | **SE** | | **95% CI** |
|  | **Fixed effects** | | | | | | | | | |
| Constant | 1.406*** | 0.121 | [1.169,1.644] | 3.558*** | 0.186 | [3.193,3.923] | 2.720*** | 0.127 | | [2.471,2.968] |
| Age | -0.154*** | 0.030 | [-0.212,-0.095] | -0.238*** | 0.040 | [-0.316,-0.160] | -0.037 | 0.031 | | [-0.098,0.023] |
| Age^2^ | 0.015 | 0.021 | [-0.027,0.056] | 0.076** | 0.287 | [0.020,0.132] | -0.004 | 0.022 | | [-0.048,0.040] |
| Girl | -0.218*** | 0.019 | [-0.255,-0.181] | -0.634*** | 0.029 | [-0.691,-0.576] | 0.043* | 0.019 | | [0.005,0.081] |
| *Ethnicity (Ref: White)* |  |  |  |  |  |  |  |  | |  |
| Mixed | -0.083 | 0.057 | [-0.196,0.029] | -0.021 | 0.088 | [-0.194,0.152] | -0.068 | 0.058 | | [-0.182,0.047] |
| Indian | -0.041 | 0.067 | [-0.173,0.091] | 0.155 | 0.104 | [-0.049,0.359] | 0.064 | 0.069 | | [-0.071,0.199] |
| Pakistani or Bangladeshi | -0.067 | 0.051 | [-0.168,0.033] | 0.307*** | 0.080 | [0.151,0.463] | 0.484*** | 0.053 | | [0.381,0.587] |
| Black | -0.556*** | 0.059 | [-0.671,-0.441] | -0.516*** | 0.091 | [-0.694,-0.338] | -0.281*** | 0.060 | | [-0.399,-0.163] |
| Other | -0.137 | 0.089 | [-0.311,0.037] | 0.062 | 0.136 | [-0.205,0.329] | 0.349*** | 0.090 | | [0.173,0.526] |
| (Easy) temperament | -0.026*** | 0.002 | [-0.029,-0.023] | -0.029*** | 0.002 | [-0.034,-0.024] | -0.035*** | 0.002 | | [-0.038,-0.032] |
| Mother is university-educated | -0.327*** | 0.025 | [-0.377,-0.278] | -0.761*** | 0.039 | [-0.838,-0.684] | -0.176*** | 0.026 | | [-0.226,-0.125] |
| Two-parent family | -0.207*** | 0.023 | [-0.252,-0.162] | -0.351*** | 0.034 | [-0.417,-0.285] | -0.062* | 0.024 | | [-0.109,-0.015] |
| SED | 0.183*** | 0.011 | [0.161,0.206] | 0.178*** | 0.016 | [0.146,0.209] | 0.110*** | 0.012 | | [0.087,0.134] |
| SED*age | -0.028*** | 0.004 | [-0.035,-0.020] | -0.005 | 0.005 | [-0.014,0.005] | -0.002 | 0.004 | | [-0.009,0.005] |
| SED*age^2^ | 0.014*** | 0.002 | [0.009,0.019] | -0.005 | 0.003 | [-0.011,0.001] | 0.006* | 0.003 | | [0.001,0.011] |
| ALE | -0.054 | 0.040 | [-0.134,0.025] | -0.046 | 0.056 | [-0.156,0.063] | -0.042 | 0.043 | | [-0.127,0.043] |
| ALE*age | -0.046** | 0.016 | [-0.078,-0.014] | -0.038 | 0.022 | [-0.081,0.006] | -0.026 | 0.017 | | [-0.060,0.008] |
| ALE*age^2^ | 0.009 | 0.012 | [-0.014,0.032] | 0.004 | 0.016 | [-0.028,0.035] | 0.019 | 0.012 | | [-0.005,0.044] |
| HPD | 0.191*** | 0.009 | [0.174,0.209] | 0.215*** | 0.013 | [0.190,0.240] | 0.053*** | 0.010 | | [0.034,0.072] |
| HPD*age | -0.011** | 0.003 | [-0.018,-0.005] | 0.019*** | 0.005 | [0.010,0.028] | 0.007 | 0.004 | | [-0.0005,0.014] |
| HPD*age^2^ | 0.008** | 0.002 | [0.003,0.013] | -0.002 | 0.003 | [-0.009,0.004] | 0.0002 | 0.003 | | [-0.005,0.005] |
| *Area stratum (Ref: England-advantaged)* |  |  |  |  |  |  |  |  | |  |
| England-disadvantaged | 0.221*** | 0.027 | [0.169,0.274] | 0.227*** | 0.042 | [0.146,0.309] | 0.114*** | 0.027 | | [0.061,0.168] |
| England-ethnic | 0.122** | 0.044 | [0.035,0.209] | 0.159* | 0.069 | [0.024,0.293] | 0.117* | 0.045 | | [0.028,0.206] |
| Scotland-advantaged | -0.033 | 0.042 | [-0.116,0.050] | -0.208** | 0.066 | [-0.337,-0.080] | -0.056 | 0.043 | | [-0.141,0.028] |
| Scotland-disadvantaged | 0.107* | 0.043 | [0.023,0.191] | 0.110 | 0.066 | [-0.020,0.239] | -0.022 | 0.044 | | [-0.108,0.064] |
| Northern Ireland-advantaged | -0.191*** | 0.009 | [-0.292,-0.090] | -0.312*** | 0.080 | [-0.468,-0.157] | -0.027 | 0.052 | | [-.0130,0.075] |
| Northern Ireland-disadvantaged | 0.097* | 0.043 | [0.014,0.181] | -0.053 | 0.066 | [-0.183,0.076] | 0.053 | 0.044 | | [-0.034,0.075] |
| Wales-advantaged | -0.006 | 0.048 | [-0.100,0.088] | -0.037 | 0.075 | [-0.183,0.109] | -0.081 | 0.049 | | [-0.177,0.015] |
| Wales-disadvantaged | 0.190*** | 0.035 | [0.121,0.259] | 0.270*** | 0.054 | [0.163,0.376] | 0.076* | 0.036 | | [0.006,0.146] |
|  | **Random effects** | | | | | | | |  |  |
| Level 2 (child) |  |  |  |  |  |  |  |  | |  |
| Between-child intercept variance | 1.069*** | 0.018 | [1.034,1.104] | 2.740*** | 0.041 | [2.660,2.822] | 1.060*** | 0.019 | | [1.023,1.097] |
| Between-child slope variance | 0.090*** | 0.003 | [0.084,0.097] | 0.118*** | 0.006 | [0.108,0.129] | 0.053*** | 0.003 | | [0.047,0.060] |
| Between-child intercept/slope variance covariance | -0.139*** | 0.005 | - | 0.097*** | 0.010 | - | 0.102*** | 0.005 | | - |
| Level 1 (occasion) |  |  |  |  |  |  |  |  | |  |
| Between-occasion variance | 1.094*** | 0.014 | [1.068,1.122] | 1.938*** | 0.025 | [1.890,1.987] | 1.280*** | 0.016 | | [1.249,1.313] |

*Note*: **p < .05, **p < .01, ***p < .001.*  *N* is not 16,916 because we did not impute missing values on the SDQ dependent variables. SED = Socio-economic disadvantage; ALE = Adverse life events; HPD = Harsh parental discipline.
